# Stability of HIB-Cul3 E3 ligase adaptor HIB Is Regulated by Self-degradation and Availability of Its Substrates

**DOI:** 10.1038/srep12709

**Published:** 2015-08-12

**Authors:** Zizhang Zhou, Congyu Xu, Ping Chen, Chen Liu, Shu Pang, Xia Yao, Qing Zhang

**Affiliations:** 1 State Key Laboratory of Pharmaceutical Biotechnology and MOE Key Laboratory of Model Animals for Disease Study, Model Animal Research Center of Nanjing University, Nanjing, 210061, China

## Abstract

The HIB-Cul3 complex E3 ligase regulates physiological homeostasis through regulating its substrate stability and its activity can be modulated by changing HIB abundance. However, regulation of HIB remains elusive. Here we provide evidence that HIB is degraded through the proteasome by Cul3-mediated polyubiquitination in K48 manner in *Drosophila*. Strikingly, HIB is targeted for degradation by itself. We further identify that three degrons (^52^LKSS^56^T, ^76^LDEE^80^S and ^117^MESQ^121^R) and K185 and K198 of HIB are essential for its auto-degradation. Finally, we demonstrate that HIB-Cul3 substrates, Ci and Puc, can effectively protect HIB from HIB-Cul3-mediated degradation. Taken together, our study indicates that there is an exquisite equilibrium between the adaptor and targets to achieve the tight control of the HIB, which is essential for maintaining suitable Hh and JNK signaling. And the mechanism of adaptor self-degradation and reciprocal control of the abundance between adaptor and its substrates is also applied to BTB-Cul3 E3 ligase adaptor dKeap1, dDiablo and dKLHL18.

## Highlights

1. HIB is unstable and degraded through ubiquitin/proteasome pathway
2. HIB is ubiquitinated by HIB-Cul3 E3 ligase
3. HIB N-terminal degrons and C- terminal K185 and K198 are essential for its degradation
4. Substrates of HIB-Cul3 ligase protect HIB from degradation

Most cellular processes require a strictly controlled coordination of regulatory proteins. Precise protein turnover allows cells to adapt rapidly to various internal and external stimuli^1^. Ubiquitin-mediated protein degradation by the 26S proteasome has emerged as a key mechanism to control protein turnover^2^. This process of delivering ubiquitin to substrates requires the coordinated action of three classes of enzymes: the E1 ubiquitin-activating enzyme, the E2 ubiquitin-conjugating enzyme, and the E3 ubiquitin-protein ligase^3^. While the E1 and E2 enzymes are primarily involved in activating and transferring ubiquitin through high-energy thioester bonds, E3 ligases play crucial roles in providing the specificity of substrate recognition^4^.

Ubiquitin ligases (E3) that confer the substrate specificity have been grouped into two families: the single-subunit forms including single RING-finger, U-box and HECT-domain E3s^5^, and the multi-subunit RING-finger ubiquitin ligases, including the Cullin-Rbx type^6^ and the APC E3s^7^. The SCF complex (Skp1, Cul1, F-box) is an example of a multi-subunit RING-finger ubiquitin ligase^8^. In the SCF ubiquitin ligase complex, Cul1 serves as a scaffold protein to assemble Skp1/F-box proteins with the small ring finger Rbx1^9^. F-box proteins directly contact their substrates and determine the specificity of the SCF complex. In addition to Cul1, genomes encode six additional Cullins (Cul2, Cul3, Cul4A, Cul4B, Cul5 and Cul7), most of which also form ubiquitin ligase complexes^10^. Notably, Cul3 interacts with some BTB-containing proteins and functions as a typical BTB-Cul3 E3 ligase complex^11^,^12^.

As a BTB protein, HIB (Hedgehog-induced MATH and BTB domain containing protein) bridges its C-terminal BTB domain to Cul3 to form an E3 ligase complex and specifically recruits substrates for ubiquitination and degradation with its N-terminal MATH domain^13^. HIB was first identified for its involvement in Hh pathway^13^,^14^. In *Drosophila*, *hib* is one of the target genes in Hh pathway; the expression of *hib* is directly activated by Ci, a transcription factor unique to the Hh pathway. HIB directly binds to Ci and then mediates the ubiquitination and degradation of Ci. Overexpression of HIB-Cul3 E3 ligase adaptor HIB downregulates Ci, and loss of HIB causes Ci accumulation in *Drosophila*^13^,^14^. These results indicate that one approach to modulate HIB-Cul3 E3 ligase activity is to regulate the level of its adaptor HIB.

Although HIB-Cul3 E3 ligase plays vital roles in many cellular processes, its regulation remains to be determined. Here, we show that HIB is degraded in a ubiquitin-dependent manner. Furthermore, we find that the destabilization of HIB requires an intact HIB-Cul3 complex; the adaptor HIB ubiquitinates itself for degradation. We further present that the S/T/D/E/Q-rich degrons and the lysine residues at 185 and 198 in HIB are responsible for its ubiquitination. Moreover, we illustrate that HIB-Cul3 E3 ligase substrates, such as Ci and Puc, can competitively bind HIB-Cul3 to protect HIB from degradation, suggesting that there is a fine-tuning balance of the E3 ligase and its substrates to maintain physiological homeostasis.

## Results

### HIB protein is unstable and degraded through Cul3

**Figure 1:**
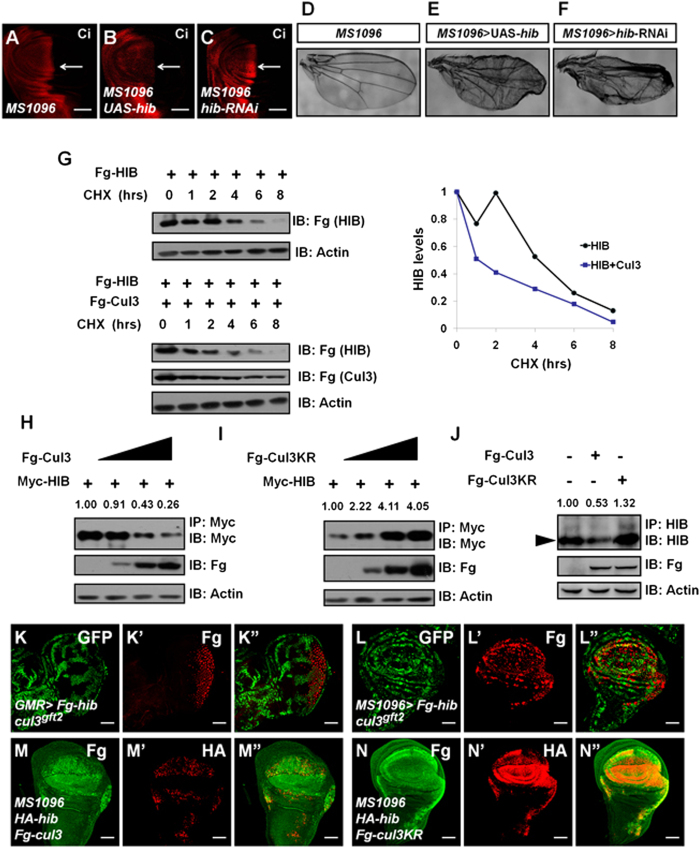
HIB protein is unstable, and its degradation is Cul3-dependent. All the wing discs shown in this study are oriented with anterior to the left and ventral at the top. *GMR-gal4* and *MS1096-gal4* lines drive the target gene expression in the posterior region of eye discs and in the pouch region of wing discs, respectively. The target gene expression with *MS1096-gal4* is typically stronger in the dorsal region of the wing pouch. *cul3*^*gft2*^ mutant clones in eye and wing discs were marked by the lack of GFP expression. (**A**–**C**) Wing discs of *MS1096* (**A**) *hib* overexpression by *MS1096* (**B**) and *hib* knockdown by *MS1096* (**C**) were stained to show Ci expression. Overexpression of *hib* decreased Ci (**B**, arrow), whereas knockdown of *hib* upregulated Ci (**C**, arrow). (**D**–**F**) Comparison of adult wing phenotypes from control flies (**D**) *hib* overexpression (**E**) and *hib* knockdown (**F**). Of note, both knockdown and overexpression of *hib* resulted in serious wing defect. (**G**) Western blots of lysates from S2 cells expressing indicated proteins and treated with CHX for the indicated time intervals. Quantification analyses of the western blots were shown on the right. Notably, Cul3 promoted HIB degradation. (**H**–**I**) Cul3 dose-dependently promoted HIB degradation (**E**) whereas Cul3KR stabilized HIB (**F**). S2 cells were transfected with 5 μg Myc-HIB and 0 μg, 0.5 μg, 1.5 μg, 4.5 μg Fg-Cul3 or Fg-Cul3KR plasmids respectively. The relative intensities of Myc-HIB bands were measured by Image J. Actin is shown as a loading control. (**J**) Cul3 decreased the endogenous HIB protein in S2 cells, while Cul3KR stabilized endogenous HIB. The relative intensities of HIB bands were measured by Image J. The arrowhead marks the band of HIB protein. Actin acts as a loading control. (**K**–**K”**) Ectopically expressed Fg-HIB with *GMR* accumulated in *cul3*^*gft2*^ mutant clones in the posterior region of eye discs. (**L**–**L”**) Ectopic-expressed Fg-HIB with *MS1096* was accumulated in *cul3*^*gft2*^ mutant clones in wing discs. (**M**–**M”**) Co-overexpression HIB and Cul3 with *MS1096* in wing discs induced HIB degradation. (**N**–**N”**) HIB was not degraded when co-overexpressed with Cul3KR. Cul3KR is a dominant negative mutant form of Cul3 with K717 mutated to R. Scale bars: 50 μm for all images.

In BTB-Cul3 E3 ligase-mediated protein degradation, BTB-Cul3 recognizes substrates through different adaptor BTB proteins^12^. In *Drosophila,* the BTB protein HIB associates with Cul3 to form an E3 ligase and recruits substrates including Ci for ubiquitination and degradation^13^,^15^. Compared with the control disc (Fig. 1A), overexpression of *hib* resulted in a decrease of Ci (Fig. 1B), whereas knockdown of *hib* increased Ci level (Fig. 1C), indicating that the regulation of HIB-Cul3 E3 ligase activity can be achieved through modulating HIB level. To test whether the level of HIB is important for maintaining physiological homeostasis, we overexpressed and knocked down *hib* in wing disc and found that either *hib* overexpression or *hib* knockdown causes abnormal adult wing phenotypes (Fig. 1D–F). To examine HIB protein stability, we used the translation inhibitor cycloheximide (CHX) to block translation and determine the stability of HIB in S2 cells. Strikingly, HIB was unstable, with a half-life of less than 4 hrs (Fig. 1G). Since HIB and Cul3 form an E3 ligase, first, we tested whether the degradation of HIB was regulated by Cul3. As shown in Fig. 1G,H, Cul3 promoted HIB destabilization in a dose-related manner. Conjugation of the ubiquitin-like molecule Nedd8 to a conserved lysine residue (K717) on Cul3 is essential for its E3 ligase activity^16^. To test whether the E3 ligase activity of Cul3 is essential for HIB destabilization, we employed a dominant-negative form of Cul3 named Cul3KR which K717 is replaced by R. Compared with Cul3, Cul3KR could stabilize HIB in S2 cells possibly by neutralizing endogenous Cul3 (Fig. 1I). We further examine the endogenous HIB protein level in S2 cells when Cul3 or Cul3KR was expressed. Cul3 expression caused HIB decreased, while Cul3KR expression resulted in an increase of HIB (Fig. 1J).

To confirm the result, we made *cul3* mutant clones and simultaneously expressed Fg-tagged HIB under the control of *GMR-gal4* in the posterior region of eye discs or *MS1096-gal4* in the pouch region of wing discs. Fg-tagged HIB accumulated in the *cul3*^gft2^ mutant clones both in eye discs (Fig. 1K–K”) and in wing discs (Fig. 1L–L”). Given that HIB protein mainly localized in nuclei^13^, the degradation of HIB occurred in nuclei (punctuate staining in the nuclei). In addition, HA-tagged HIB was degraded when Cul3 was co-expressed in wing discs (Fig. 1M–M”). Contrarily, Cul3KR expression did not result in HIB decreased (Fig. 1N–N”). All the above data verify that the degradation of HIB protein is through Cul3 and Cul3 ligase activity is essential for its function on HIB degradation.

### HIB protein degradation is via polyubiquitination and the proteasome

**Figure 2:**
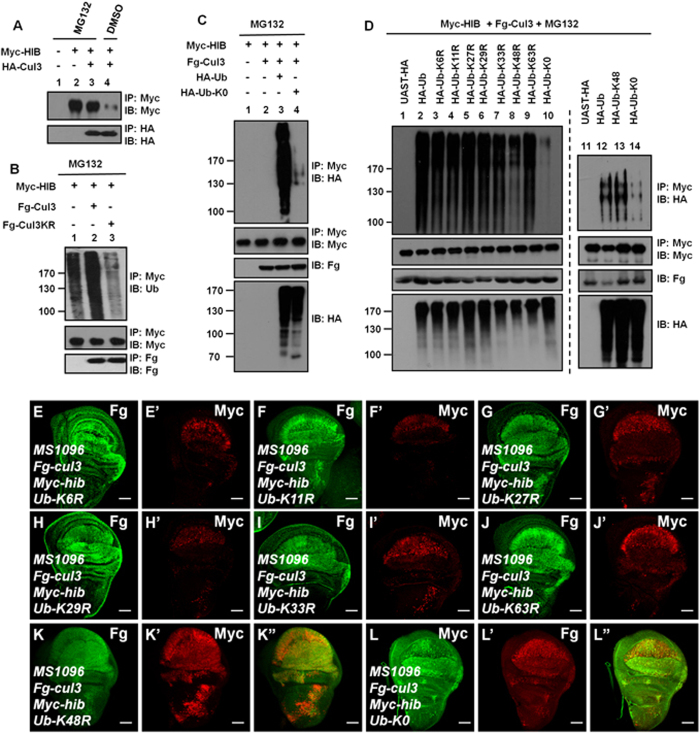
Cul3 promotes HIB K48-linked polyubiquitination. Ub-K0 is a mutant form of Ub, in which all Ks are replaced with Rs. Ub-K6R, K11R, K27R, K29R, K33R, K48R, and K63R are mutant forms of Ub with K on residue 6, 11, 27, 29, 33, 48, and 63, respectively, replaced with R. (**A**) HIB was degraded through the proteasome. In the presence of the proteasome inhibitor MG132, the level of HIB had no detectable difference when Cul3 was co-expressed. In the absence of MG132, HIB protein abundance was remarkably reduced. (**B**) Cul3 promoted HIB ubiquitination. The ubiquitination of HIB was apparently increased when Cul3 was co-expressed, whereas, the ubiquitination level was decreased when Cul3KR was co-expressed. (**C**) Cul3 induced the polyubiquitination of HIB. The ubiquitination level had a striking difference when HA-Ub or HA-Ub-K0 was co-expressed. Ub-K0 cannot form polyubiquitin chains. (**D**) Cul3 promoted K48 linkage polyubiquitination of HIB. Ubiquitination assays were carried out using different Ub mutants. (**E**–**J’**) Ub-K6R (**E’**), Ub-K11R (**F’**), Ub-K27R (**G’**), Ub-K29R (**H’**), Ub-K33R (**I’**) and Ub-K63R (**J’**) could not prevent HIB degradation induced by Cul3. (**K**–**L”**) Ub-K48R (**K’**) and Ub-K0 (**L’**) apparently attenuated Cul3-mediated HIB degradation. Scale bars: 50 μm for all images.

Cul3-mediated substrate turnover is through substrate ubiquitination and ultimately 26S proteasome degradation^17^. Likewise, treatment with the proteasome inhibitor MG132 effectively increased HIB protein level (Fig. 2A, lane3), whereas in the absence of MG132, HIB protein abundance was apparently reduced when Cul3 was co-expressed (Fig. 2A, lane4), suggesting that the degradation of HIB is likely through ubiquitination and the proteasome. To further test this possibility, we carried out a ubiquitination assay. As shown in Fig. 2B, Cul3 promoted HIB ubiquitination (lane2), whereas Cul3KR attenuated HIB ubiquitination (lane3). Without Cul3 co-transfection, the weak ubiquitination of HIB is likely through endogenous Cul3 (Fig. 2B, lane1). Taken together, these data support the assertion that Cul3 promotes the ubiquitin-dependent degradation of HIB.

Ubiquitin-mediated protein degradation can occur through monoubiquitination or polyubiquitination^18^. To test which type of ubiquitination contributes to HIB degradation, we employed Ub-K0, a mutant form of Ub, in which all seven lysine residues (K6, K11, K27, K29, K33, K48 and K63) are replaced by Arginines (Rs), preventing polyubiquitin chain formation^19^. The ubiquitination level of HIB apparently decreased when co-expressed with Ub-K0 compared with wild-type Ub (Fig. 2C), suggesting that HIB is subjected to polyubiquitination. To further characterize the Cul3-mediated Ub modification on HIB, we began with identifying the type of Ub chain linkages, as the distinct linkage of polyubiquitin chains has been implicated in determining diverse fates of substrates^20^,^21^,^22^,^23^,^24^,^25^. An ubiquitination assay was performed via several Ub mutants, in whom one of the seven Ks was replaced by R: Ub-K6R, Ub-K11R, Ub-K27R, Ub-K29R, Ub-K33R, Ub-K48R and Ub-K63R. As shown in Fig. 2D, the ubiquitination level of HIB was eliminated when Ub-K48R and Ub-K0 were used (lanes 8 and 10), whereas other mutants did not affect the ubiquitination of HIB. The Ub-K48, which harbors only one K on 48, had the similar effect on Cul3-mediated HIB ubiquitination (compare Fig. 2D lane13 with lane 12). The findings suggest that Cul3 catalyzes HIB K48-linkaged polyubiquitination. Furthermore, we generated different Ub lysine mutant transgenic flies and examined which of these mutants could attenuate HIB degradation. Compared with other Ub mutants, only Ub-K0 and Ub-K48R could effectively inhibit Cul3-mediated HIB destabilization (Fig. 2E–L”). Taken together, Cul3 promotes K48-linkages polyubiquitin modification of HIB and subsequent degradation.

### A BTB domain-containing protein is responsible for HIB degradation

**Figure 3:**
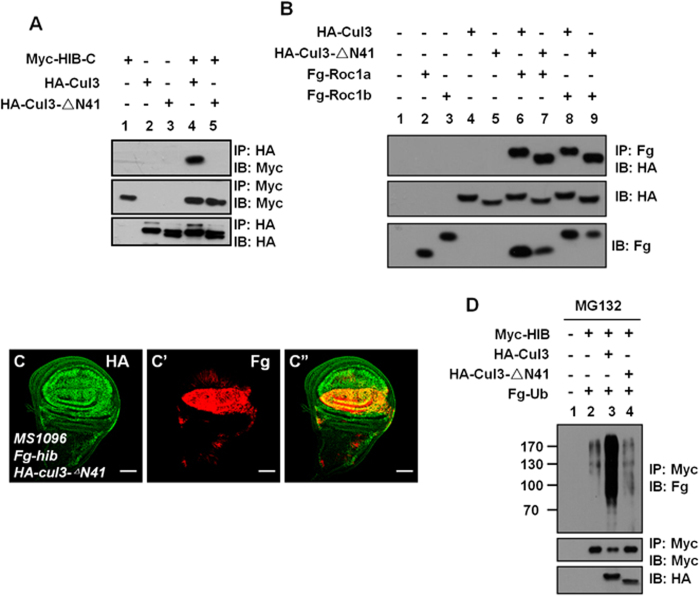
The degradation of HIB is conducted by a BTB-containing protein. (**A**) The HIB-C associated with Cul3 but not Cul3-ΔN41. HIB-C contains aa180–374 of HIB. Cul3-ΔN41 is a form of Cul3 with a deletion of amino acids from 35 to 75. (**B**) Both Cul3 and Cul3-ΔN41 interacted with Roc1a and Roc1b. (**C**–**C”**) HIB was not degraded when co-expressed with Cul3-ΔN41 (compared with Fig. 1M–M”). (**D**) Compared with Cul3, Cul3-ΔN41 did not promote HIB ubiquitination. Scale bars: 50 μm for all images.

Increasing evidence suggests that BTB proteins function as substrate-specific adaptors of Cul3 ligases^12^,^26^. To test whether Cul3-mediated HIB ubiquitination occurs through a BTB-containing protein, we constructed a truncated form of Cul3 (Cul3- ΔN41) with a deletion of 41 amino acids in the N-terminus (from Trp^35^ to Tyr^75^). These 41 residues include a hydrophobic helix, H2, which is involved in binding to the BTB domain^27^. As expected, wild-type Cul3 could bind to the C-terminal fragment (HIB-C) (Fig. 3A, lane 4), whereas deletion of the N-terminal 41 residues apparently disrupted this interaction (Fig. 3A, lane 5). We further found that Cul3-ΔN41 could bind the ring box proteins, such as Roc1a and Roc1b (Fig. 3B), indicating that the deletion of 41 amino acids did not affect Cul3 interaction with an E2 ubiquitin-conjugating enzyme. If Cul3-dependent HIB degradation is through a BTB-containing protein, Cul3-ΔN41 will not induce HIB ubiquitination and degradation. Consistent with this hypothesis, HIB was not degraded when Cul3-ΔN41 was co-expressed in the wing discs (Fig. 3C–C”). The ubiquitination assay also showed that Cul3, but not Cul3-ΔN41, promoted HIB ubiquitination (compared Fig. 3D lane3 with lane4). These data indicate that a BTB-containing protein is account for HIB ubiquitination and degradation.

### HIB ubiquitination is mediated by HIB-Cul3 E3 ligase

**Figure 4:**
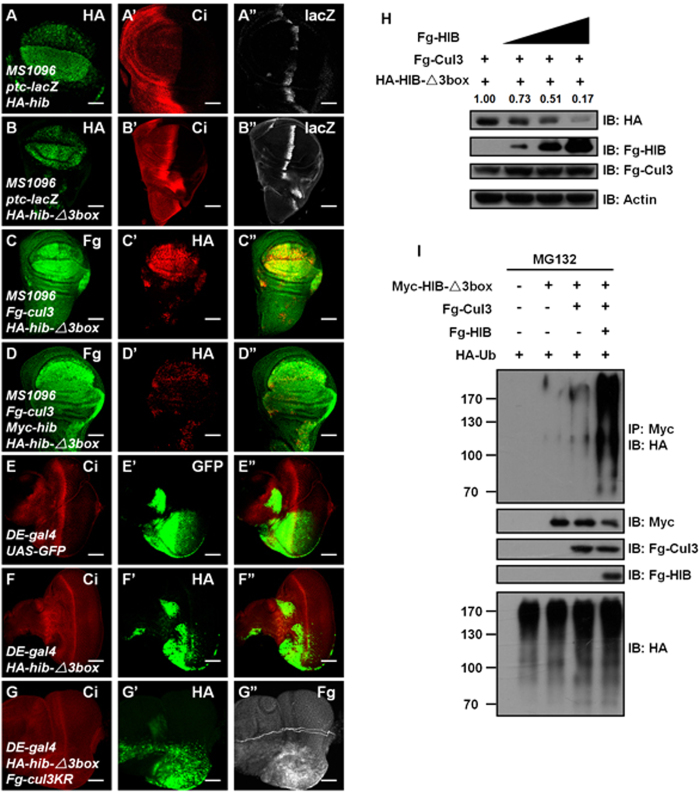
HIB is degraded by itself. (**A-A”**) A wing disc expressing HA-*hib* by MS1096 was stained to show HA (green), Ci (red) and *ptc*-lacZ (white) expression. Overexpression of *hib* decreased Ci and *ptc*-lacZ levels. (**B-B”**) 3-box domain (aa299–330) deletion destroyed HIB E3 ligase function (compared with Fig. 4A–A”). (**C**–**C”**) HIB-Δ3box failed to be degraded by Cul3 in wing discs. The level of HIB-Δ3box had a subtle decrease near the A/P boundary, where the endogenous *hib* is expressed. (**D**–**D”**) HIB-Δ3box was apparently degraded when co-expressed with HIB and Cul3. (**E**–**E”**) GFP marked the expression region of *DE-gal4* in eye discs. (**F**–**F”**) HIB-Δ3box was destabilized in the posterior region of eye discs, where the endogenous *hib* is ubiquitously expressed. (**G**–**G”**) Co-expression of Cul3KR could effectively stabilize HIB-Δ3box protein in the posterior of eye discs. (**H**) HIB-Cul3 E3 ligase promoted HIB-Δ3box degradation. The relative intensities of HA-HIB-Δ3box bands were measured by Image J. Actin acts as a loading control. (**I**) Co-expression of HIB and Cul3 promoted the ubiquitination of HIB-Δ3box in S2 cells. Scale bars: 50 μm for all images.

The above results suggest that a BTB-containing protein conjugates with Cul3 to promote HIB degradation. Given that HIB is a BTB protein and forms a complex with Cul3 as an E3 ligase^13^,^15^, it was prudent to first test whether HIB degradation is mediated by itself. We employed a mutant form of HIB, in which the 3-box domain (Cul3-associated domain; from Val^299^ to Thr^330^) was deleted^28^. The 3-box domain sits adjacent to the BTB domain and plays a decisive role for HIB association with Cul3 to allow for HIB-Cul3 E3 ligase activity^29^,^30^. Deletion of the 3-box domain abolishes the association between BTB and Cul3^29^ and destroys the E3 ligase function of HIB, as monitored by the hindered capacity to degrade the well-known target, Ci (compare Fig. 4B,B” with Fig. 4A–A”).

We found that HIB-Δ3box failed to be degraded when Cul3 was co-expressed in wing discs (Fig. 4C–C”). However, co-expression of exogenous wild-type HIB and Cul3 could induce an apparent decrease of HIB-Δ3box in wing discs (Fig. 4D–D”). Furthermore, HIB-Δ3box protein was mildly decreased both near A/P boundary in wing discs (Fig. 4B,C’) and the posterior region in eye discs (Fig. 4F–F”), where the endogenous *hib* is expressed. Consistent with this observation, co-expression of the dominant-negative Cul3KR apparently upregulated the HIB-Δ3box protein level in the posterior of eye discs (compare Fig. 4G–G” with 4F–F”). Furthermore, we found that HIB promoted HIB-Δ3box degradation in a dose-dependent manner in S2 cells (Fig. 4H). We also test the ubiquitination level of HIB-Δ3box protein, and found that only co-expression of HIB-Cul3 increased the ubiquitination of HIB-Δ3box (Fig. 4I). These data demonstrate that HIB is targeted for ubiquitination through HIB-Cul3 E3 ligase.

### The N-terminal degrons and the C-terminal Lys^185^ or Lys^198^ are essential for HIB degradation

**Figure 5:**
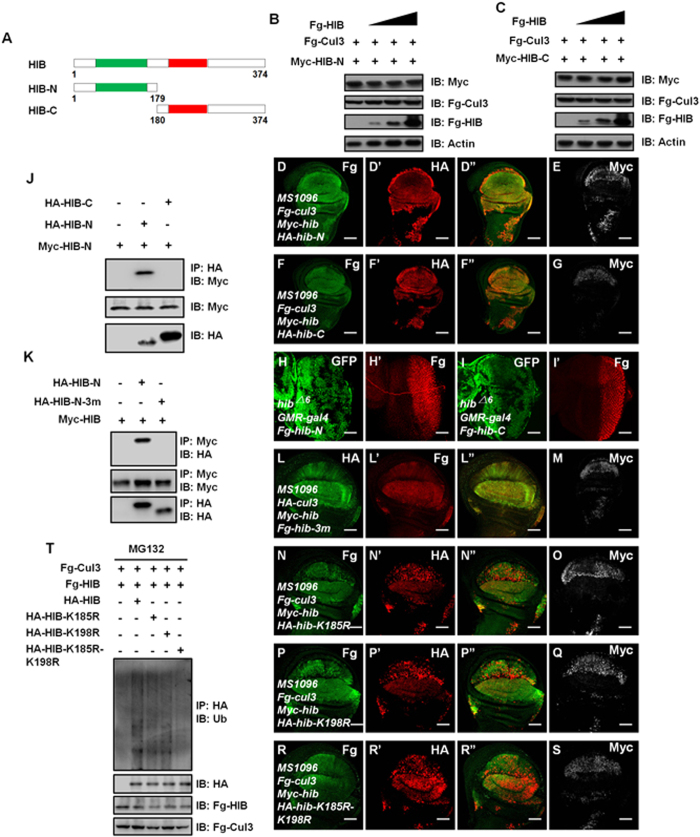
The recognition degrons and K185 or K198 on HIB are essential for it ubiquitination and degradation. (**A**) Schematic drawings show the domains in HIB and its truncated fragments used in subsequent assays. Green and red bars denote the MATH and BTB domains of HIB. (**B**–**C**) HIB-Cul3 E3 ligase did not promote the degradation of HIB-N (**B**) and HIB-C (**C**) in S2 cells. Actin acts a loading control. (**D**–**E**) Wing discs expressing HA-*hib*-N plus Myc*-hib* and Fg*-cul3* were stained to show Fg (green), HA (red) and Myc (white). HIB-Cul3 failed to degrade HIB-N. (**F**–**G**) Wing discs expressing HA-*hib*-C plus *hib*-*cul3* were stained to show Fg (green), HA (red) and Myc (white). HIB-Cul3 failed to degrade HIB-C. (**H**–**H’**) Fg-HIB-N protein levels did not increase in *hib* mutant clones marked by the lack of GFP. Fg-HIB-N overexpression was driven by *GMR*-gal4 in the posterior region of eye discs. (**I**–**I’**) HIB-C protein did not accumulate in *hib* mutant clones in eye discs. (**J**) HIB-N pulled down HIB-N, not HIB-C. (**K**) HIB interacted with HIB-N, but not HIB-N-3m. HIB-N-3m is a mutant form of HIB-N that three potential S/T/D/E/Q-rich motifs are replaced by As. (**L**–**M**) HIB-Cul3 did not degrade HIB-3m. (**N**–**O**) Wing discs expressing HA-*hib*-K185R plus Myc*-hib* and Fg*-cul3* were stained to show Fg (green), HA (red) and Myc (white). K185 mutation to R (HA-HIB-K185R) partially blocked HIB destabilization caused by HIB-Cul3 E3 ligase. (**P**–**Q**) K198 mutation to R (HA-HIB-K198R) partially prevented HIB from degradation induced by HIB-Cul3. (**R**–**S**) The degradation of HIB was totally blocked when both K185 and K198 were replaced by Rs (HA-HIB-K185R-K198R). (**T**) Western blots of immunoprecipitates (top) or lysates (bottom three panels) from S2 cells expressing indicated proteins and treated with MG132. HIB ubiquitination levels induced by HIB-Cul3 E3 ligase were decreased when K185 and K198 were replaced by Rs on HIB. Scale bars: 50 μm for all images.

HIB contains two functional domains: the MATH domain at the N-terminus for substrate recognition and binding, and the BTB domain for association with Cul3 (Fig. 5A)^13^,^15^. To study the roles of the MATH domain and the BTB domain for HIB degradation, we employed two truncated constructs: Myc-HIB-N, comprising the MATH domain and its flanking sequence (aa 1 to 179); and Myc-HIB-C, including BTB domain and its flanking sequence (aa 180 to 374) (Fig. 5A)^13^,^15^. As shown in Fig. 5B,C, Myc-HIB-N and Myc-HIB-C failed to be degraded by HIB-Cul3 E3 ligase. We also analyzed the stability of HIB-N and HIB-C in wing discs when co-expressed with the HIB-Cul3 complex and found that these two truncated HIB proteins were not degraded by HIB-Cul3 E3 ligase (Fig. 5D–G). To further confirm these results, we generated *hib* mutant clones in eye discs and simultaneously expressed Fg-tagged HIB-N or HIB-C by *GMR*-gal4 in the posterior region of eye discs. Consistently, there were no any detectable protein accumulations *hib* mutant clones (Fig. 5H–I’). These results support that the intact structure of HIB is essential for its degradation.

Neither the N-terminal nor the C-terminal fragments alone is sufficient for HIB degradation, suggesting that the HIB-Cul3 recognition degron(s) and the polyubiquitin chain attachment site(s) located in different fragments. HIB, acting as an E3 ligase, recognizes the substrates by its N-terminal fragment, and the C-terminus was account for association with Cul3^13^. The Co-IP experiments determined that HIB-N interacted with HIB-N, not HIB-C (Fig. 5J), indicating that the HIB-Cul3 recognition sites sit in HIB-N. Given HIB binds substrates through some S/T/D/E/Q-rich degrons^15^,^29^, we found three potential S/T/D/E/Q-rich motifs in the MATH domain of HIB: ^52^LKSS^56^T, ^76^LDEE^80^S and ^117^MESQ^121^R. When mutated these S/T/D/E/Q-rich motifs to generate HIB-N-3m that all the amino acids were replaced by alanines, we found that HIB could bind HIB-N, but not HIB-N-3m (Fig. 5K). To further check whether these motifs are important for HIB destabilization, we generated a Fg-*hib*-3m transgenic fly, and tested the stability of this mutant form in wing discs. HIB-Cul3 E3 ligase failed to promote Fg-HIB-3m degradation (Fig. 5L,M). These data indicate that the substrate HIB is recognized by HIB-Cul3 E3 ligase via its S/T/D/E/Q-rich degrons in MATH domain.

The previous data showed that the MATH domain of HIB did not self-interact, but HIB-N could form a dimer/oligomer^15^, suggesting the flanking sequences of MATH play important roles for HIB-N self-association. To test this possibility, various truncated constructs were generated (Figure S1A). Consistently, HA-tagged HIB-MATH did not pull down Fg-tagged HIB-MATH (Figure S1B). However, both HIB-N-1 (contains MATH domain and upstream sequence) and HIB-N-2 (contains MATH domain and downstream sequence) could form homodimer (Figures S1C,D), suggesting that the upstream and downstream sequences of MATH domain are essential for HIB-N self-interaction.

According to the above information, the polyubiquitin chains likely attached to the C-terminus of HIB. To determine this possibility, we fused one of the degrons (LKSST) to the N-terminus of HIB-C to construct LKSST-HIB-C chimeric protein. When expressed in S2 cells, HIB-N pulled down LKSST-HIB-C, but not HIB-C (Figure S1E), indicating that LKSST-HIB-C may be a substrate of HIB-Cul3 ligase. Consistently, HIB induced LKSST-HIB-C degradation in a dose-dependent manner (Figure S1F). Furthermore, we found that the lysine residues at 185 and 198 had important role for HIB ubiquitination. The mutation of K185 or K198 to R partially attenuated HIB destabilization induced by HIB-Cul3 ligase (Fig. 5N–Q). Furthermore, the degradation was almost totally blocked when both K185 and K198 were replaced by Rs (Fig. 5R,S). Through ubiquitination assay, the ubiquitination level of HIB decreased when either K185 or K198 was replaced by R (Fig. 5T). These data suggest that the polyubiquitin chains are attached to K185 and K198, and that the ubiquitin-tagged HIB is transported into the proteasome for degradation.

### Self-ubiquitination is applied for some other BTB-containing E3 ligases

**Figure 6:**
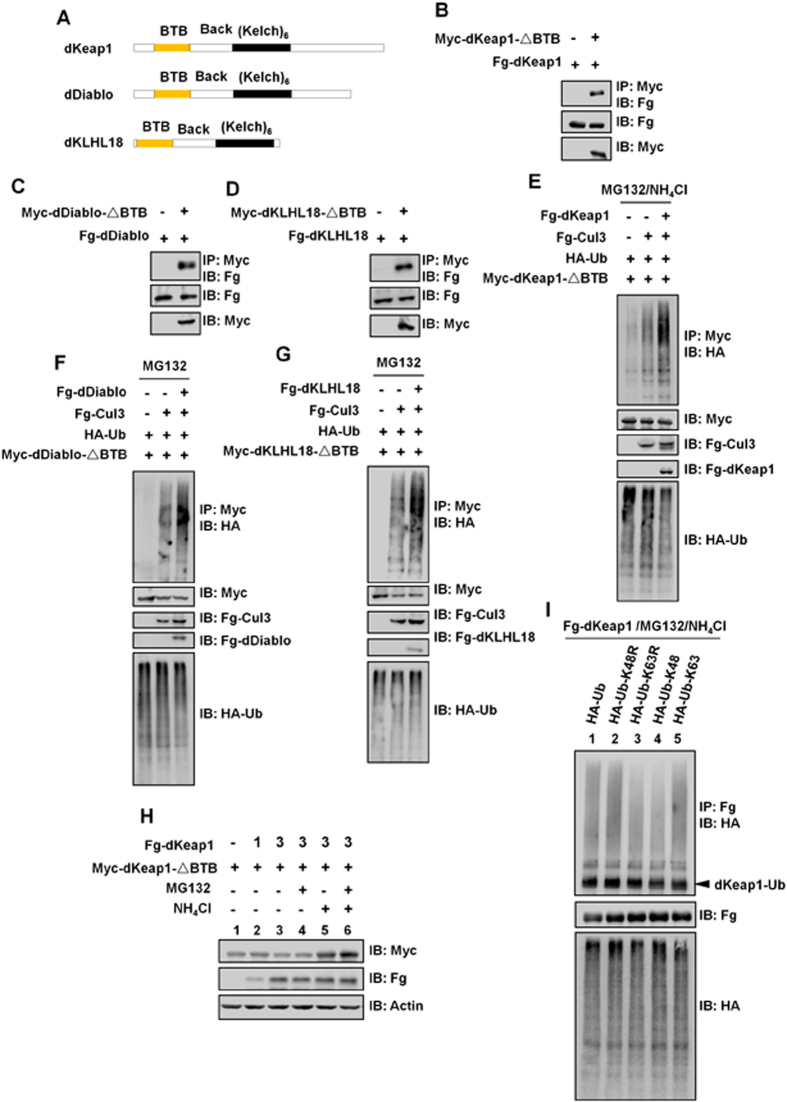
Some BTB-containing adaptors are regulated by auto-ubiquitination. (**A**) Schematic drawings show the domains in dKeap1, dDiablo and dKLHL18. Yellow and black bars denote the BTB and tandem Kelch domains. (**B**) dKeap1-ΔBTB pulled down dKeap1. (**C**) dDiablo-ΔBTB binds dDiablo. (**D**) dKLHL18-ΔBTB pulled down dKLHL18. (**E**) dKeap1-Cul3 promoted the ubiquitination of dKeap1-ΔBTB. The transfected cells were treated with NH_4_Cl for 16hrs and MG132 for 4hrs prior cell harvesting. (**F)** dDiablo-Cul3 promoted the ubiquitination of dDiablo-ΔBTB. (**G**) dKLHL18-Cul3 promoted the ubiquitination of dKLHL18-ΔBTB. From F to G, the transfected S2 cells were treated with MG132 for 4hrs before cell harvesting. (**H)** dKeap1 promoted dKeap1-ΔBTB degradation in a dose-dependent manner. The indicated cells were treated with NH_4_Cl for 16hrs or/and MG132 for 4hrs prior cell harvesting. (**I**) dKeap1 was modified by K63-linked polyubiquitin chains. The transfected cells were treated with NH_4_Cl for 16 hrs and MG132 for 4hrs prior cell harvesting.

BTB-domain-containing proteins commonly form homodimers using BTB domain^15^,^29^,^31^,^32^. Consistently, the HIB-C indeed formed a homodimer/oligomer (Figure S1G). However, except for that, we found HIB-N also formed a dimer (Fig. 5J and Figures S1C,D). To address whether this BTB/BTB-domain-independent self-interaction was unique to HIB, we tested other BTB-containing E3 ligases including dKeap1, dDiablo and dKLHL18 (Fig. 6A). Through Co-IP experiments, dKeap1-ΔBTB could pull down dKeap1; dDiablo-ΔBTB pulled down dDiablo; dKLHL18-ΔBTB pulled down dKLHL18 (Fig. 6B–D). In addition, dKeap1-ΔBTB, dDiablo-ΔBTB and dKLHL18-ΔBTB could form homodimers, respectively (Figures S2B–D). These results indicate that BTB/BTB-independent self-association is likely to be applied for these adaptors. Except for BTB domain, dKeap1 harbors Back and six repeated Kelch domains (Fig. 6A and Figure S2A). We found that dKeap1-Back did not form a dimer (Figure S2E), and dKeap1-Back also did not bind dKeap1-Kelch (Figure S2F). However, Co-IP experiments showed that dKeap1-Kelch formed dimer (Figure S2G). These results suggest that the BTB/BTB-independent self-association of dKeap1 is mediated by the Kelch domain. We further found that this case was applied for dDiablo and dKLHL18 (Figures S2H–M). Although dKeap1, dDiablo and dKLHL18 harbor Kelch domain, the amino acid composition in Kelch domain differs significantly. To test whether the Kelch domains form heterodimer, we expressed different Kelch domains in S2 cells. Through Co-IP assay, Kelch domain exclusively formed homodimer (Figure S2N). The previous data revealed that dKeap1, dDiablo and dKLHL18 associated with Cul3 using BTB domains to form E3 ligases^33^,^34^,^35^,^36^. We examined the ubiquitination of dKeap1-ΔBTB and found that dKeap1-Cul3 overexpression promoted its ubiquitination (Fig. 6E), suggesting that dKeap1 was likely to be a substrate of dKeap1-Cul3 E3 ligase. In addition, we uncovered that this self-ubiquitination was also applied for dDiablo and dKLHL18 (Fig. 6F,G).

We further investigated whether the degradation of dKeap1 was mediated by dKeap1 itself and found that dKeap1 promoted dKeap1-ΔBTB destabilization in a dose-dependent manner (Fig. 6H, lane1–3 and Figure S2, lane1–3). The degradation of dKeap1-ΔBTB was inhibited when treated with lysosome inhibitor NH_4_Cl (Fig. 6H, lane5 and lane 6), not proteasome inhibitor MG132 (Fig. 6H, lane 4), suggesting that the destabilization of dKeap1-ΔBTB was through lysosome. In fact, the previous data have revealed that mammalian Keap1 is degraded by autophagy^37^,^38^,^39^. We further identified that dKeap1 occurred K63 linkage, not K48 linkage polyubiquitination (Fig. 6I). The K63-linked polyubiquitin chain modifications commonly targeted the substrates for vesicle-mediated trafficking or degradation^40^,^41^,^42^. Taken together, these results suggest that the ubiquitination and degradation of dKeap1 is mediated, at least in part, by dKeap1-Cul3 E3 ligase.

### Substrates efficiently prevent HIB from degradation through competitive association with HIB-Cul3 ligase

**Figure 7:**
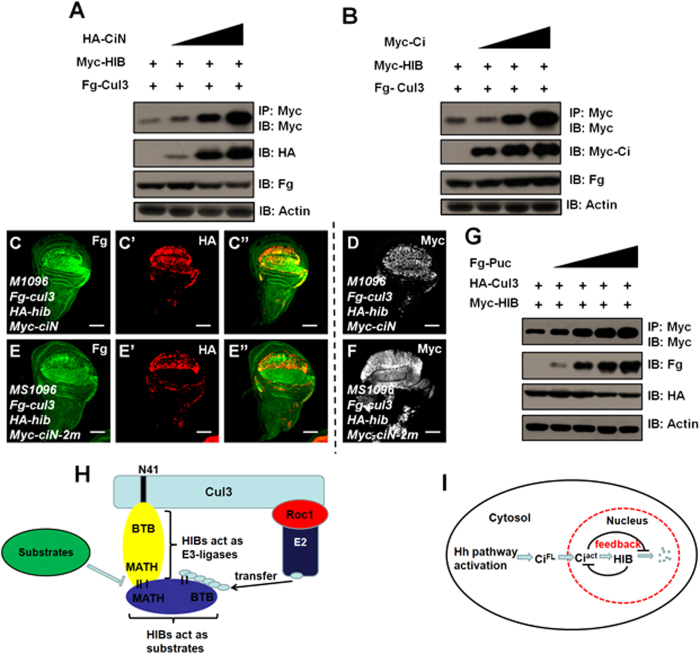
Substrates prevent HIB from degradation. CiN (aa1-440) is a truncated form of Ci that contains two HIB binding motifs (^368^PEQPSSTS^376^G and ^379^AQVEADSASSQLS^392^D). CiN-2m is a mutant form of CiN, in which both binding motifs are mutated. The expression of CiN and CiN-2m were shown in (**D**) and (**F**) respectively. (**A**) CiN prevented HIB from degradation in a dose-dependent manner. (**B**) Ci inhibited Cul3-induced HIB degradation in a dose-dependent manner. (**C**–**D**) HIB degradation through Cul3 was blocked when overexpressed with CiN in wing discs. (**E**–**F**) The inhibitory effect of CiN disappeared when both HIB binding motifs on CiN were mutated. (**G**) Puc could efficiently protect HIB from destabilization. (**H**) HIB protein associates with Cul3 through its BTB domain. HIB acting as an adaptor of HIB-Cul3 E3 ligase recruits dissociative HIB protein through the MATH domain, thus HIB-Cul3 promotes dissociative HIB ubiquitination and degradation. Substrates competitively bind the adaptor HIB, thus preventing dissociative HIB degradation. (**I**) The physiological significance of HIB auto-regulated degradation. In the presence of Hedgehog protein, Ci is transported to the nucleus and acts as a transcription activator. Ci promotes *hib* expression; conversely, HIB degrades Ci through ubiquitinating Ci. When Ci level is low, excess HIB is degraded in an auto-regulated ubiquitination. In contrast, when the HIB level is low, Ci will protect HIB from degradation. This delicate feedback loop allows appropriate Ci and HIB levels to remain in the nucleus. Scale bars: 50 μm for all images.

Both HIB and Ci are ubiquitinated and subsequently destabilized by HIB-Cul3 E3 ligase. We sought to determine whether they competitively bind to the HIB-Cul3 complex. Since Ci N-terminus (CiN) can bind HIB to result in Ci degradation through HIB-Cul3 E3 ligase^13^,^15^. We performed assays in S2 cells and found that CiN substantially stabilized HIB in a dose-dependent manner (Fig. 7A). The full-length Ci (Ci^FL^) also attenuated Cul3-mediated HIB degradation (Fig. 7B). To further verify this result in wing discs, we employed CiN because Ci^FL^ overexpression stimulated endogenous *hib* transcription^13^. Co-expressed CiN could efficiently stabilize HIB (Fig. 7C,D). In contrast, mutating the HIB binding sites in CiN led to the loss of its protection of HIB degradation (Fig. 7E,F). Next, it was essential to investigate whether this protection is broadly applicable to other substrates. As expected, another well-known substrate, JNK phosphatase puckered (Puc), also prevented HIB from destabilization (Fig. 7G). In addition, we found the dKeap1 substrate, dNrf2, also attenuated dKeap1-ΔBTB degradation mediated by dKeap1 (Figure S3A, lane4-6). The corresponding substrates (dDsh and CG10324)^33^,^35^ also protected dDiablo and dKLHL18 from self-degradation (Figures S3B,C). We further found that the substrates of Keap1-, dDiablo- and dKLHL18-Cul3 E3 ligases could block their cognate Kelch-Kelch self-association (Figures S3D–F). These results suggest that the reciprocal control of the abundance between adaptor and its substrates is applied for dKeap1, dDiablo and dKLHL18.

## Discussion

HIB-Cul3 E3 ligase recognizes and ubiquitinates substrates, culminating in their degradation by the 26S proteasome^13^,^15^. In *Drosophila*, HIB influences many cellular processes through targeting its substrates, including Hedgehog pathway transcription factor Ci and JNK pathway Puc. Both knockdown and overexpression of *hib* in wing cause serious wing abnormalities (Fig. 1D–F), suggesting that the strict monitoring of HIB level is crucial. Self-degradation of the adaptor HIB will change its abundance, limiting the portion of Cul3 in Cul3 pool are motivated to couple with HIB to form HIB-Cul3 E3 ligase and thus limiting HIB-Cul3 E3 ligase activity. This idea is supported by data showing that either the overexpression or knockdown of HIB alone is enough to modulate HIB-Cul3 E3 ligase activity as determined by alterations in the level of its substrate Ci.

The well-defined substrates Ci and Puc effectively protect HIB from degradation through competitive interacting with HIB-Cul3 ligase, raising the possibility that some other substrates may have similar functions. This protection could play an important role for keeping the equilibria between the adaptor and targets. Ultimately, these equilibria tightly monitor the homeostasis of corresponding signaling pathways. In Hedgehog pathway, for example, in the presence of Hh, Ci accumulates in the nucleus and promotes HIB synthesis. In turn, HIB degrades Ci through HIB-Cul3 E3 ligase. When the Ci protein level is low, excess HIB is degraded by itself through an auto-catalytic mechanism. However, when HIB protein reaches a very low level, Ci competitively associates with HIB-Cul3 and prevents HIB from further destabilization. Through this elegant regulation, Ci and HIB form a fine-tuning feedback loop that strictly monitors Hh pathway activity (Fig. 7H,I).

Although the previous data have been demonstrated that F-box adaptors in SCF E3 ligase are degraded by an auto-catalytic mechanism^43^,^44^, the regulation of other adaptors in Cul-containing E3s, including Cul2 and Cul3, has to be determined. Our findings uncovered that a BTB-containing adaptor HIB in HIB-Cul3 E3 ligase was regulated via auto-ubiquitination. Except for HIB, we also found the some other BTB-containing adaptors, including dKeap1, dDiablo and dKLHL18 were regulated by auto-ubiquitination. Therefore, it is interesting to investigate more BTB-containing adaptors to test whether they are modulated by self-ubiquitination. Here we should emphasize that even above BTB-containing adaptors are modulated by self-ubiquitination, but maybe their degradation goes through distinct ways. It is possible that they are destabilized by either proteasome or lysosome affected by their ubiquitination manners. Given that most Cullin proteins (Cul1, Cul2, Cul3, Cul4, Cul5 and Cul7) together with adaptors to form E3 ligases, it is fruitful to determine whether other adaptors are regulated by auto-catalytic mechanism.

Except for HIB, some Kelch domain-containing proteins are also conjugated with Cul3 to form various Kelch-Cul3 E3 ligases. For Kelch domain-containing adaptors, albeit their primary sequences of amino acids are different, they form beta-propellers which consist of several Kelch repeats^45^. The previous findings revealed that beta-propeller subunits could form a dimer through the aromatic residues on the dimer interface and charged side chains^46^. Our data showed that the Kelch domains from dKeap1, dDiablo and dKLHL18 exclusively formed homo-dimers, suggesting that beta-propeller may favor of forming homo-dimer, however, distinct primary sequences may endue their specificity for homo-dimerization and substrate recognizing. The advantage of adaptor self-degradation likely provides a fine-tuning mechanism to efficiently switch adaptors and achieve different BTB-Cul3 E3 ligase activities according to stimuli.

SPOP is an evolutionary homolog of HIB in vertebrates, and it shares extensive amino acid sequence identity with HIB^13^. As reported, SPOP recruits several targets to the SPOP-Cul3 E3 ligase for ubiquitination and subsequent degradation, including polycomb group BMI^47^, anti-apoptotic protein Daxx^48^ and breast cancer metastasis suppressor1 (BRMS1)^49^. Recently, it has been reported that the mutation or deficiency of SPOP closely links to many tumors, including glioma^50^, prostate cancers^51^, breast cancers^49^ and endometrial tumors^52^, suggesting that the regulation of SPOP is important for homeostasis. A previous study also demonstrated that SPOP could undergo ubiquitination via Cul3^53^. It is fruitful to investigate whether SPOP is ubiquitinated by SPOP-Cul3 E3 ligase.

## Methods

### Constructs, Mutants, and Transgenes

The constructs for expression in S2 cells were generated as follows: the Fg-HIB, Fg-Cul3, Fg-Cul3KR, Myc-HIB, HA-Cul3, Myc-HIB-C, Myc-HIB-N, HA-CiN and Myc-Ci constructs have been previously described^13^,^15^. To construct Fg-Roc1a, HA-Roc1b, Fg-Ub, HA-Ub, Fg-dKeap1, Fg-dDiablo, Fg-dKLHL18 HA-dNrf2 and Fg-Puc plasmids, we amplified the corresponding cDNA fragments, and then cloned them into pUAST-3×Fg or pUAST-3×HA vectors. The point mutant constructs used in this study were generated using PCR-based site-directed mutagenesis at the background of Fg-Ub or Fg-HIB. The truncated constructs were generated by inserting the corresponding coding sequences into the pUAST-3×HA or pUAST-6×Myc vectors. *cul3*^*gft2*^, *DE-gal4, GMR-gal4*, *MS1096-gal4, eyflp*, *hs-flp* and *UAS-GFP* were from Bloomington Stock Center (Flybase). *UAS-Fg-cul3, UAS-Fg-cul3KR, UAS-HA-hib, UAS-Fg-hib, UAS-Fg-hib-N, UAS-Fg-hib-C, UAS-Myc-ciN, UAS-Myc-ciN-2m, ptc-lacZ* and *hib*^*Δ6*^ have been described^13^,^15^. *UAS-HA-ub-K6R, UAS-HA-ub-K11R, UAS-HA-ub-K27R, UAS-HA-ub-K29R, UAS-HA-ub-K33R, UAS-HA-ub-K48R, UAS-HA-ub-K63R, UAS-HA-ub-K0, UAS-HA-cul3, UAS-HA-cul3*-*ΔN41, UAS-Myc-hib, UAS-HA-hib-N, UAS-HA-hib-C, UAS-HA-hib-Δ3box, UAS-HA-hib-3m, UAS-HA-hib-K185R, UAS-HA-hib-K198R* and *UAS-HA-hib-K185R-K198R,* were generated by injection of corresponding vectors into *Drosophila* embryos according to the methods described previously^54^. The parental strain for all germline transformations was *w*^*1118*^. All stocks were maintained and raised under standard conditions.

### Immunostaining and microscopy

For immunostaining of imaginal discs, third-instar larvae were dissected in PBS and fixed in freshly made 4% formaldehyde in PBS buffer at room temperature for 20 min, then washed three times with buffer PBT (PBS, 0.1% Triton X-100). To avoid non-specific interaction, larvae were blocked with PBTA (PBT, 10% BSA) for 30 min, next, were incubated overnight with needed primary antibodies in PBTA at 4 °C , then washed with PBT for three times and incubated with appropriate fluorophore-conjugated secondary antibody dilutions in PBTA for 2 hr at room temperature. After washed for three times in PBT, imaginal discs were dissected and mounted in 80% glycerol. Images were captured with FV10-ASW 2.0 Olympus confocal microscope. Primary antibodies were used at the following dilutions: mouse anti-HA (F7) (1:200; Santa Cruz); rabbit anti-HA (Y11) (1:200; Santa Cruz); mouse anti-Myc (9E10) (1:200; Santa Cruz); rabbit anti-Fg (PA1-984B) (1:200; Thermo Scientific); mouse anti-β-Gal (G8021) (1:500; Sigma) and Rat anti-Ci (2A1) (1:50; DSHB).

### Generating mutant clones

Clones of mutant cells were generated by the FLP-FRT system as described^55^. The genotypes of the generated clones were as follows: *cul3* clones in wing discs expressing *UAS-Fg-hib*: *hs-flp, MS1096; cul3*^*gft2*^ *FRT40/hs-Myc-GFP FRT40; UAS-Fg-hib/+*; *cul3* clones in eye discs expressing *UAS-Fg-hib*: *eyflp; cul3*^*gft2*^ *FRT40/hs-Myc-GFP FRT40; GMR-gal4/UAS-Fg-hib*; *hib* clones in eye discs expressing *UAS-Fg-hib-N* or *UAS-Fg-hib-C*: *eyflp; GMR-gal4/UAS-Fg-hib-N or UAS-Fg-hib-C; hib*^*Δ*6^ *FRT82/hs-Myc-GFP FRT82*. For GFP expression, the 3^rd^ larvae were heat shocked at 37 °C for 1.5 hrs and then kept at room temperature for another 1.5 hrs and subjected to dissection according to standard protocols.

### Cell culture, Transfection, Immunoprecipitation and Western blot

S2 cells were maintained at 25 °C in Schneider’s *Drosophila* Medium (S9895, Sigma) supplemented with 10% FBS (Gibco) and 1% penicillin/streptomycin (P0781, Sigma). Transfection was performed using calcium phosphate according to the manufacturer’s instructions (Invitrogen). An ub-gal4 plasmid was co-transfected with pUAST expression vectors for all experiments. 48 hrs after transfection, cells were harvested for immunoprecipitation and western blot analysis with standard protocols (described in *Molecular Cloning*). The primary antibodies used were mouse anti-HA (F7) (1:2500; Santa Cruz); rabbit anti-HA (Y11) (1:2500; Santa Cruz), mouse anti-Myc (9E10) (1:2500; Santa Cruz); mouse anti-Fg (1:2500; Sigma); mouse anti-Ub (P4D1) (1:1000; Santa Cruz); rabbit anti-HIB (1:1000; ABclonal Technology) and mouse anti-Actin (A00702) (1:5000: Genscript). Rabbit anti-HIB antibody was generated in rabbit with full length HIB protein as antigen (from ABclonal Technology). After incubation with HRP-coupled secondary antibodies (goat anti-mouse diluted 1:10000, Abmax; goat anti-rabbit diluted 1:10000, Jackson ImmunoResearch), the blots were visualized using a chemiluminescent detection kit (GE healthcare).

### Protein Stability Assays

S2 cells were plated in 10-cm dishes and transfected with plasmids after18–24 hrs. After another 24 hrs, the cells were transferred into 6-well cell culture plates at equivalent densities. Cells were treated with 20 μg/ml CHX (Calbiochem) for the indicated times before harvesting. After western blots, the band intensity was measured by Image J.

### Ubiquitination Assays

S2 cells were transiently transfected with the indicated combinations of expression vectors. Four hours before cells harvesting, MG132 (Calbiochem) was added to the media at a final concentration of 10 μM. The ubiquitination assays were then carried out based on the previously described protocol^13^. Briefly, cells were lysed with denaturing buffer (1% SDS, 50 mM Tris-base, pH 7.5, 0.5 mM EDTA, and 1 mM DTT) and incubated at 100 °C for 5 min. The lysates were then diluted 10-fold with regular lysis buffer and subjected to immunoprecipitation and Western blot analysis.

## Additional Information

**How to cite this article**: Zhou, Z. *et al.* Stability of HIB-Cul3 E3 ligase adaptor HIB Is Regulated by Self-degradation and Availability of Its Substrates. *Sci. Rep.* **5**, 12709; doi: 10.1038/srep12709 (2015).

## Supplementary Material

Supplementary Information
