# Supplementary material for: Stability of HIB-Cul3 E3 ligase adaptor HIB Is Regulated by Self-degradation and Availability of Its Substrates
Source: Sci Rep. 2015 Aug 12;5:12709. doi: 10.1038/srep12709 (PMC4533009; doi:10.1038/srep12709)

Supplementary information

Stability of HIB-Cul3 E3 ligase adaptor HIB Is Regulated by Self-degradation and Availability of Its Substrates

Zizhang Zhou [1], Congyu Xu [1], Ping Chen [1], Chen Liu [1], Shu Pang [1], Xia Yao[1] and Qing Zhang [1,] *

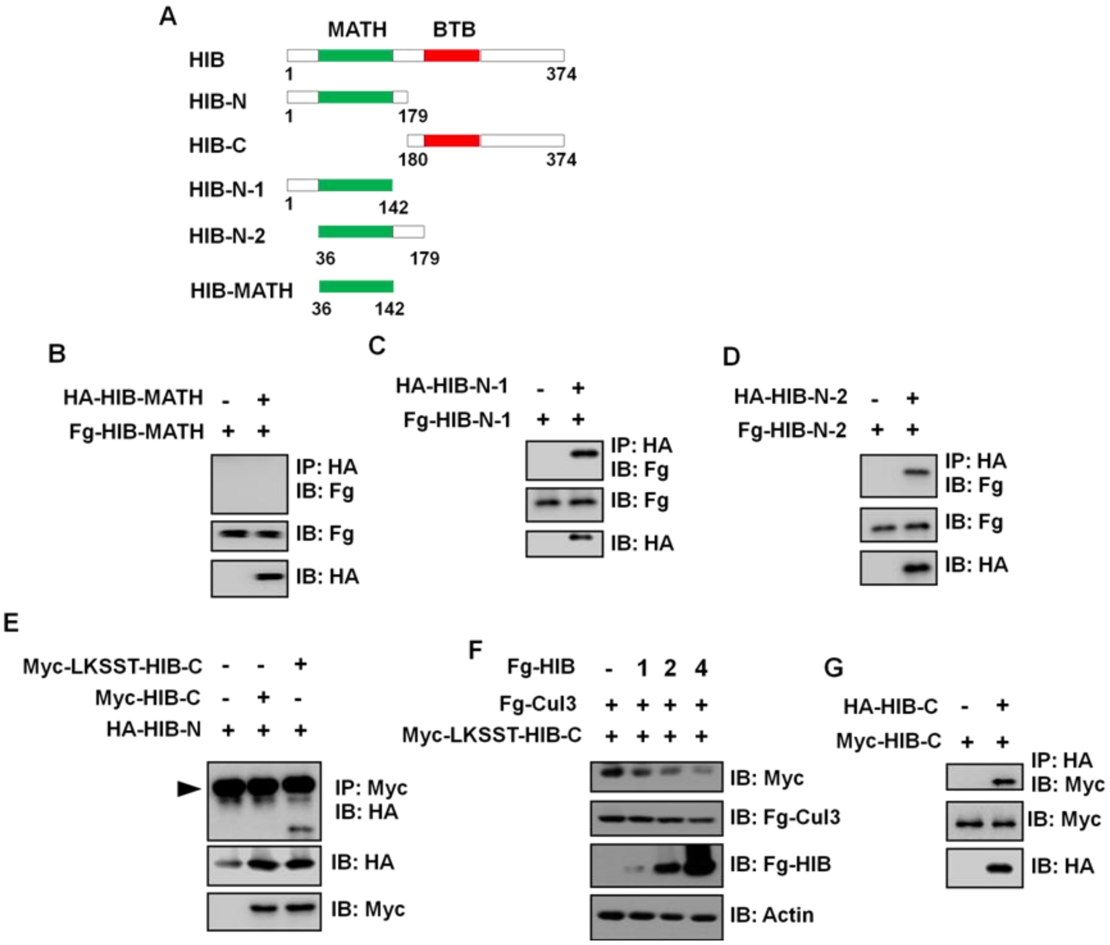

Figure S1. HIB can form a dimer through its N-terminus or C-terminus

(A) Schematic drawings show the domains in HIB and its truncated fragments

used in subsequent assays. Green and red bars denote the MATH and BTB domains of Ci.

(B) HIB-MATH did not form a dimer.

(C-D) HIB-N-1 and HIB-N-2 could form a homodimer.

(E) HIB-N interacted with LKSST-HIB-C, but not HIB-C. The arrowhead indicates IgG band.

(F) HIB-Cul3 promoted LKSST-HIB-C degradation in a dose-dependent manner.

(G) HIB-C formed a dimer.

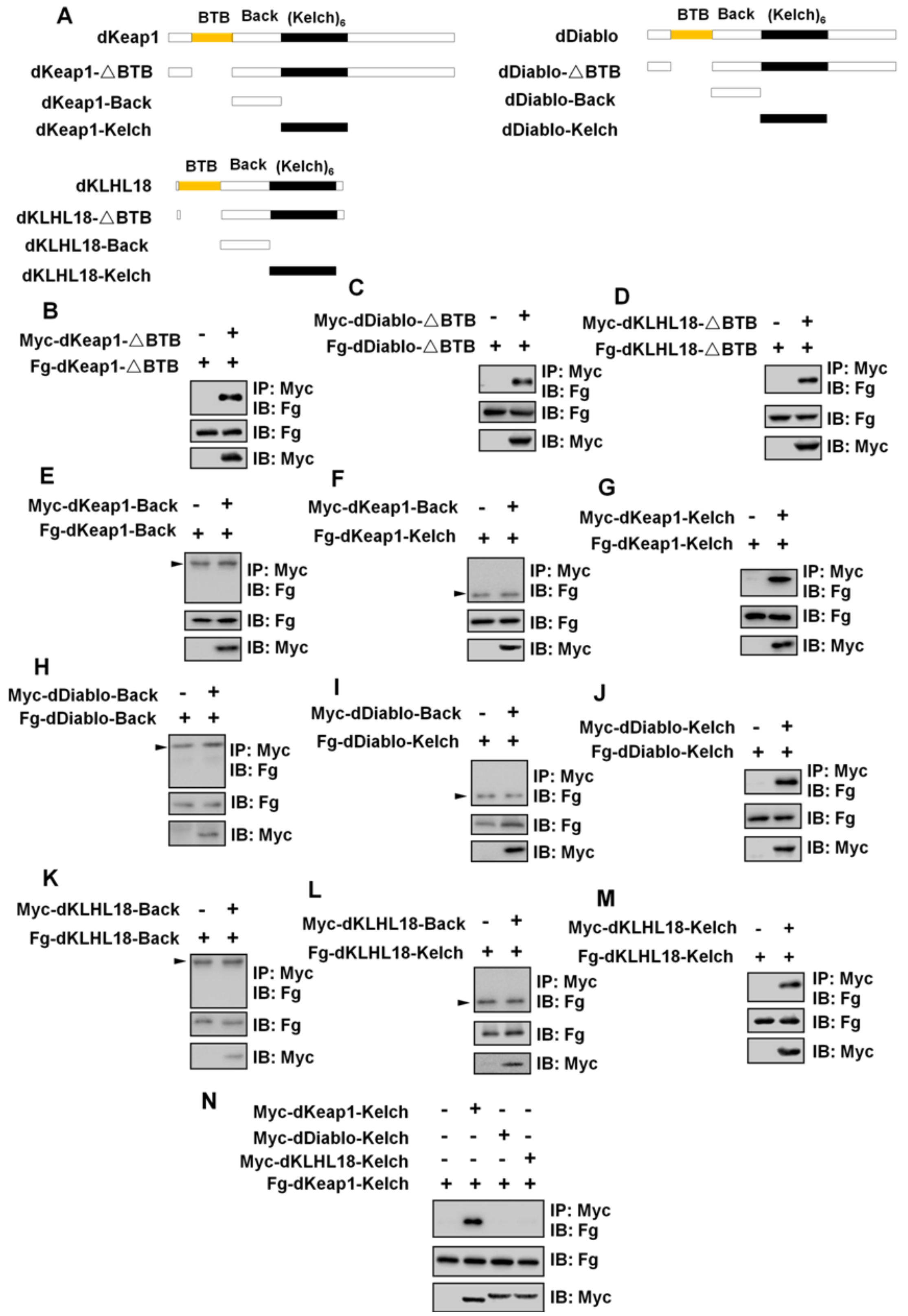
A
BTB Back (Kelch)6
dKeap1
dKeap1-△BTB
dKeap1-Back
dKeap1-Kelch
dDiablo
dDiablo-△BTB
dDiablo-Back
dDiablo-Kelch
dKLHL18
dKLHL18-△BTB
dKLHL18-Back
dKLHL18-Kelch
B
Myc-dKeap1-△BTB - +
Fg-dKeap1-△BTB + +
C
Myc-dDiablo-△BTB - +
Fg-dDiablo-△BTB + +
D
Myc-dKLHL18-△BTB - +
Fg-dKLHL18-△BTB + +
E
Myc-dKeap1-Back - +
Fg-dKeap1-Back + +
F
Myc-dKeap1-Back - +
Fg-dKeap1-Kelch + +
G
Myc-dKeap1-Kelch - +
Fg-dKeap1-Kelch + +
H
Myc-dDiablo-Back - +
Fg-dDiablo-Back + +
I
Myc-dDiablo-Back - +
Fg-dDiablo-Kelch + +
J
Myc-dDiablo-Kelch - +
Fg-dDiablo-Kelch + +
K
Myc-dKLHL18-Back - +
Fg-dKLHL18-Back + +
L
Myc-dKLHL18-Back - +
Fg-dKLHL18-Kelch + +
M
Myc-dKLHL18-Kelch - +
Fg-dKLHL18-Kelch + +
N
Myc-dKeap1-Kelch - + - -
Myc-dDiablo-Kelch - - + -
Myc-dKLHL18-Kelch - - - +
Fg-dKeap1-Kelch + + + +
IP: Myc
IB: Fg
IB: Fg
IB: Myc

Figure S2. dKeap1, dDiablo and dKLHL18 can form homodimer through the Kelch domain

(A) Schematic drawings show the domains in dKeap1, dDiablo and dKLHL18 and their truncated fragments used in subsequent assays.

(B) dKeap1-ΔBTB formed a dimer.

(C) dDiablo-ΔBTB formed a dimer.

(D) dKLHL18-ΔBTB formed a dimer.

(E) dKeap1-Back did not form a dimer. Arrowhead indicates IgG.

(F) dKeap1-Kelch did not pull down dKeap1-Back. Arrowhead indicates IgG.

(G) dKeap1-Kelch could form a dimer.

(H) dDiablo-Back did not form a dimer. Arrowhead indicates IgG.

(I) dDiablo-Kelch did not pull down dDiablo-Back. Arrowhead indicates IgG.

(J) dDiablo-Kelch could form a dimer.

(K) dKLHL18-Back did not form a dimer. Arrowhead indicates IgG.

(L) dKLHL18-Kelch did not pull down dKLHL18-Back. Arrowhead indicates IgG.

(M) dKLHL18-Kelch could form a dimer.

(N) dKeap1-Kelch was only pulled down by dKeap-Kelch, not by dDiablo-Kelch and dKLHL18-Kelch.

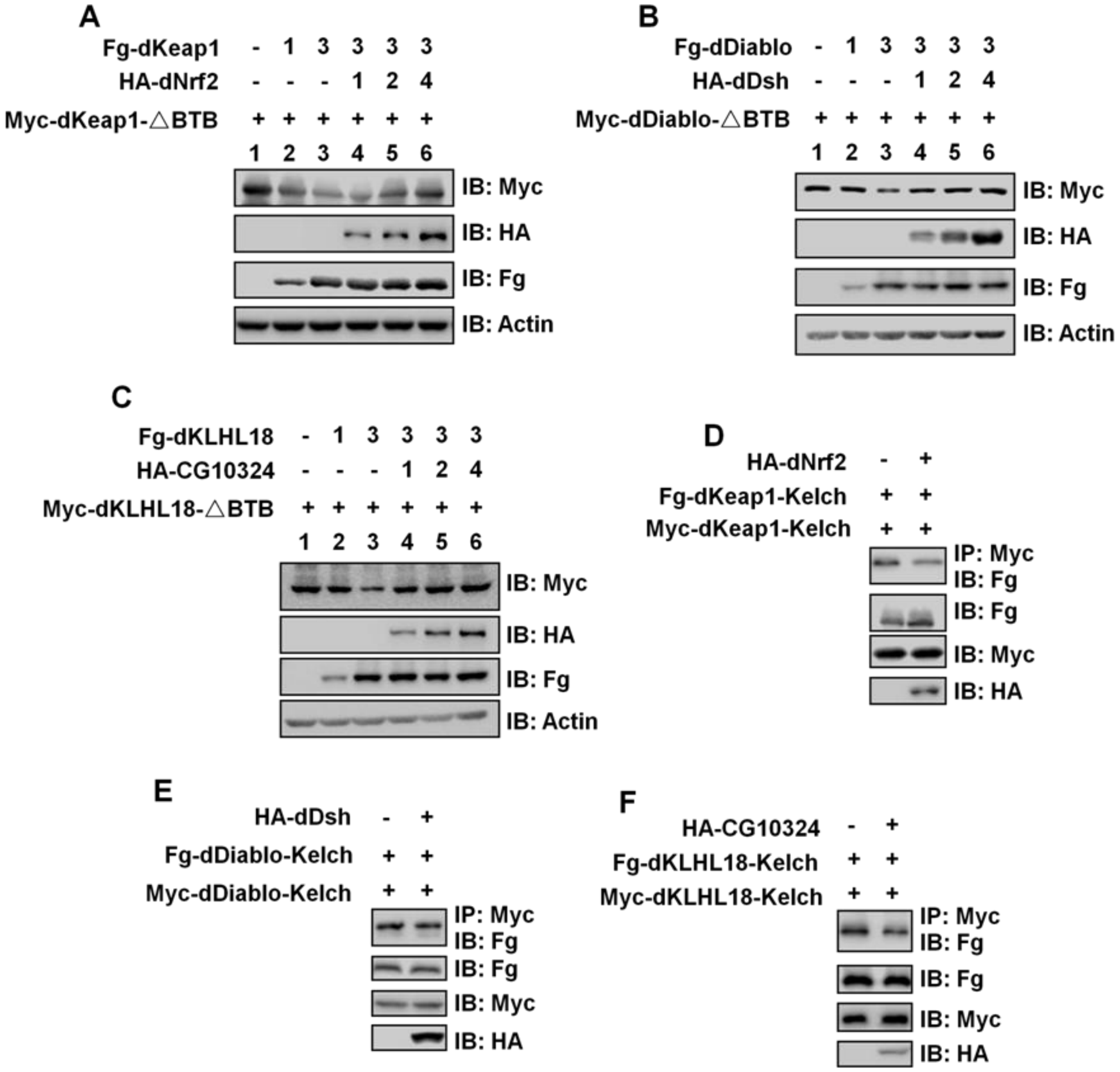

Figure S3. Substrates prevent self-degradation of dKeap1, dDiablo and dKLHL18

(A) dNrf2 prevented dKeap1-mediated dKeap1-ΔBTB degradation.

(B) dDsh prevented dDiablo-mediated dDiablo-ΔBTB degradation.

(C) CG10324 prevented dKLHL18-mediated dKLHL18-ΔBTB degradation.

From A to C, actin acts as a loading control.

(D) dNrf2 decreased dKeap1-Kelch self-association.

(E) dDsh decreased dDiablo-Kelch self-association.

(F) CG10324 decreased dKLHL18-Kelch self-association.

Figure. 1G

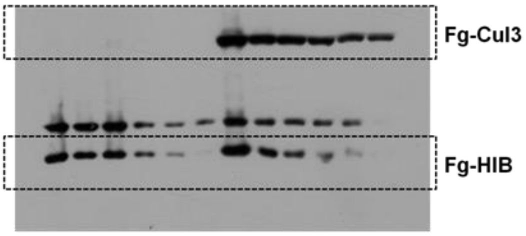

Figure. 1H

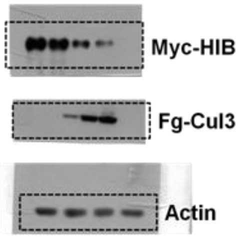

Figure. 1I

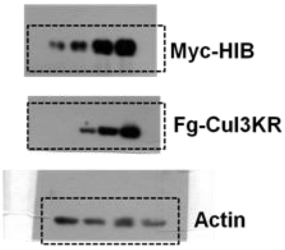

Figure. 1J

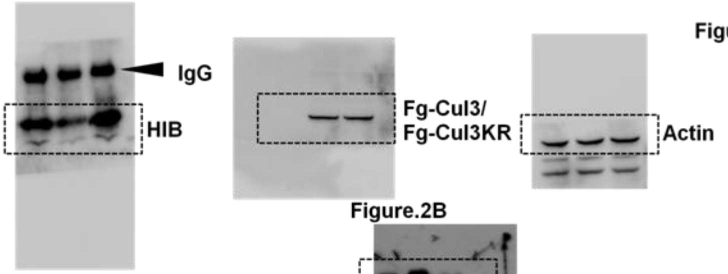

Figure.2A

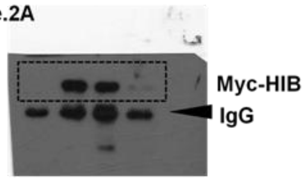

Figure.2B

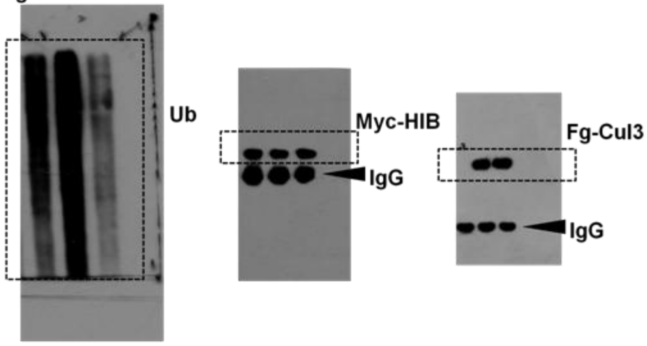

Figure.2A

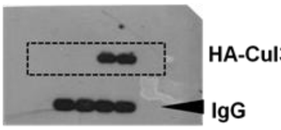

Figure.2C

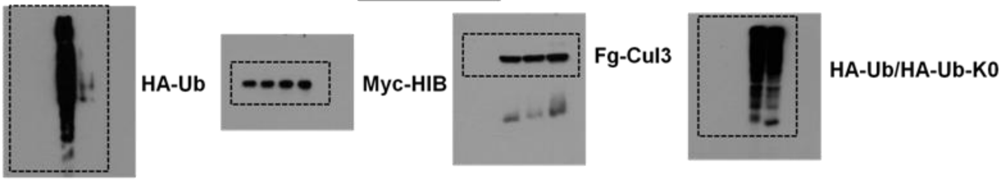

Figure.2D

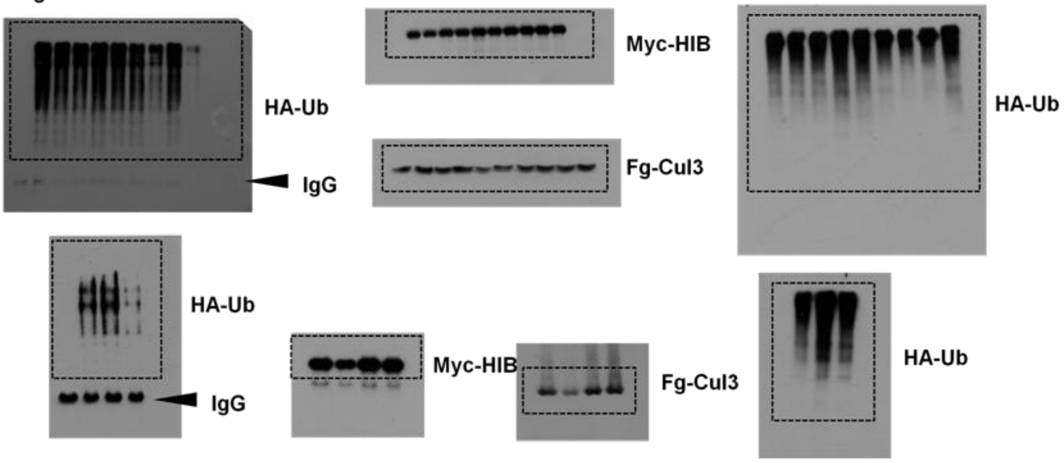

Figure. 3A

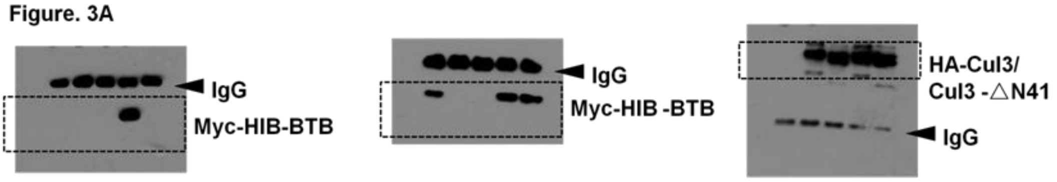

Figure. 3B

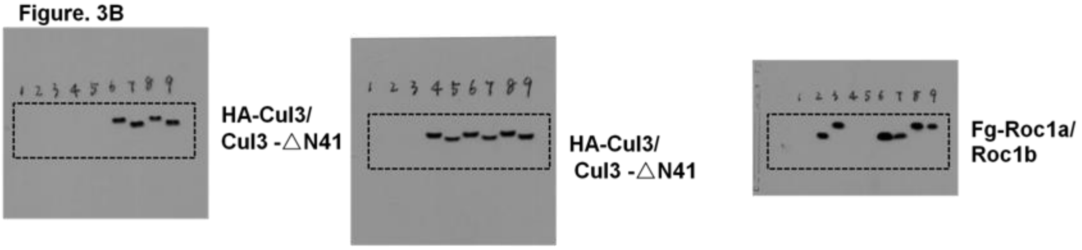

Figure. 3D

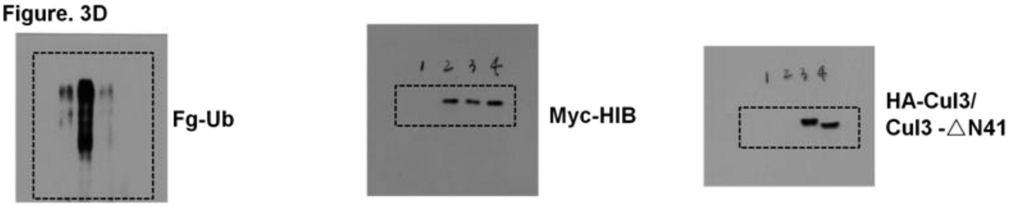

Figure.4I

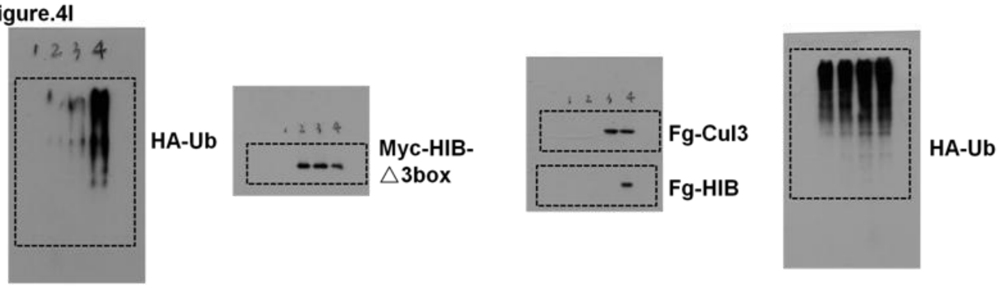

Figure. 5T

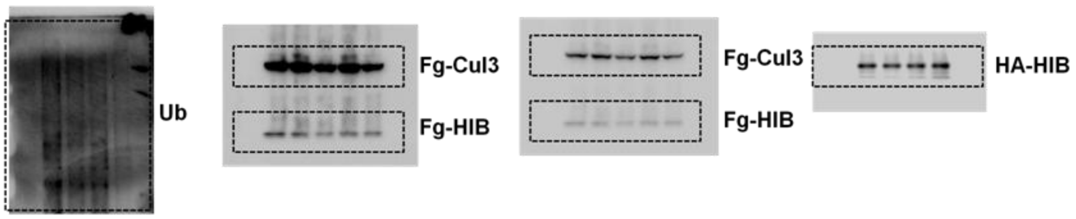

Figure. 5K

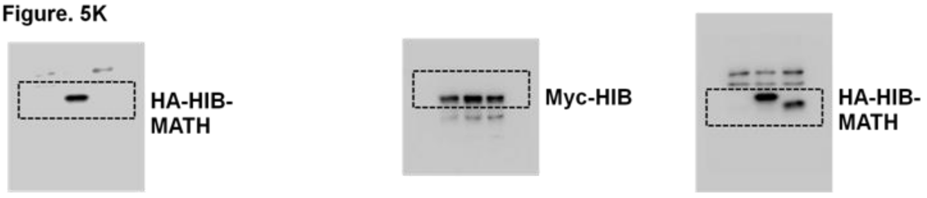

Figure.6B

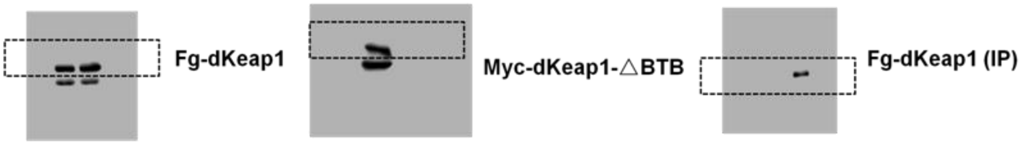

Figure.6C

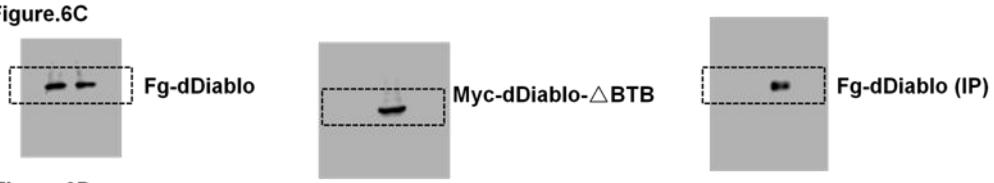

Figure.6D

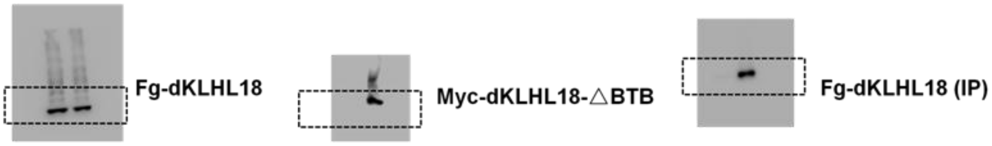

Figure.6E

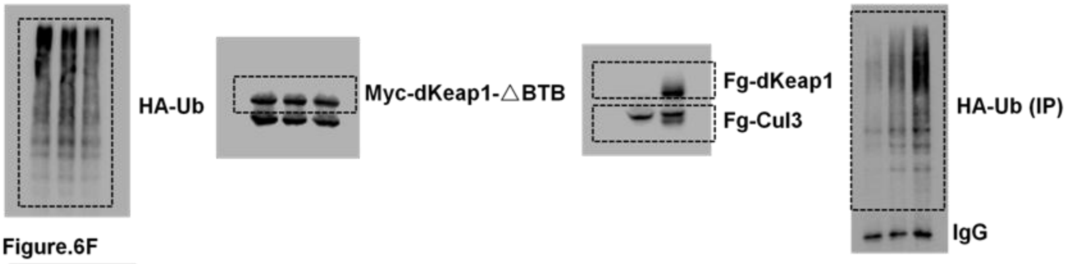

Figure.6F

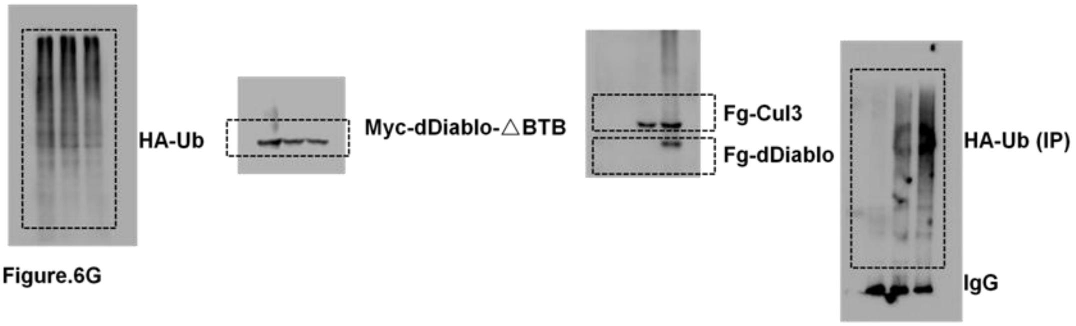

Figure.6G

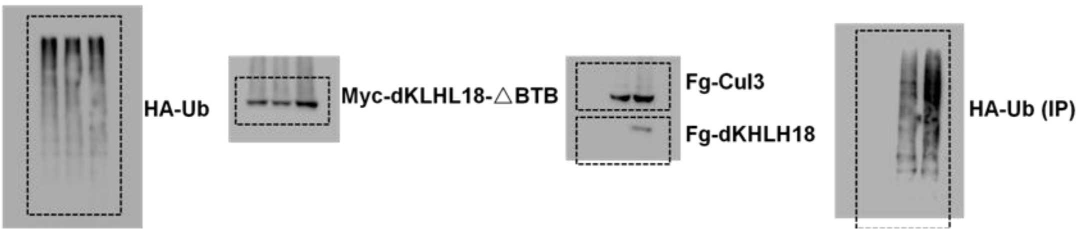

Figure.6H

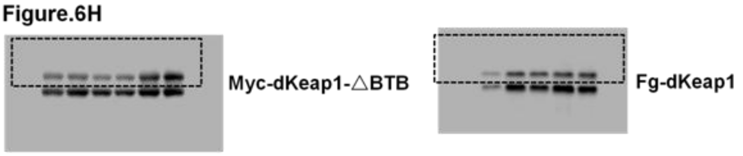

Figure.6I

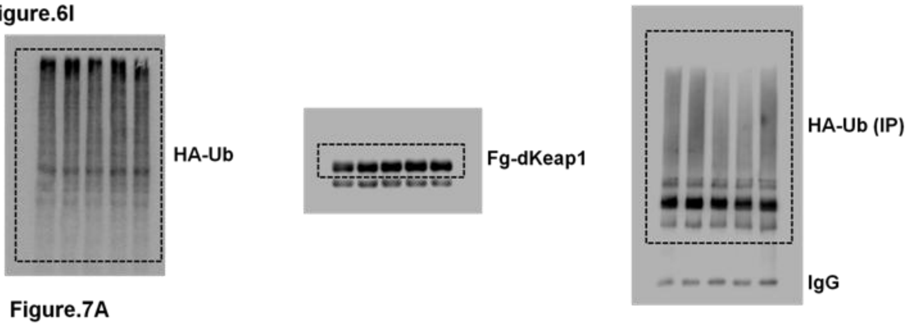

Figure.7A

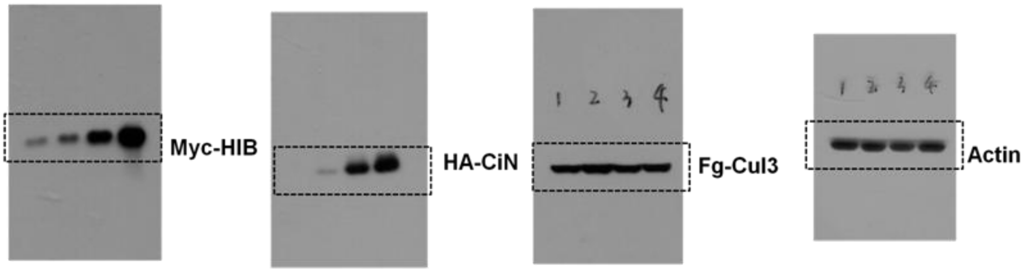

Figure.7B

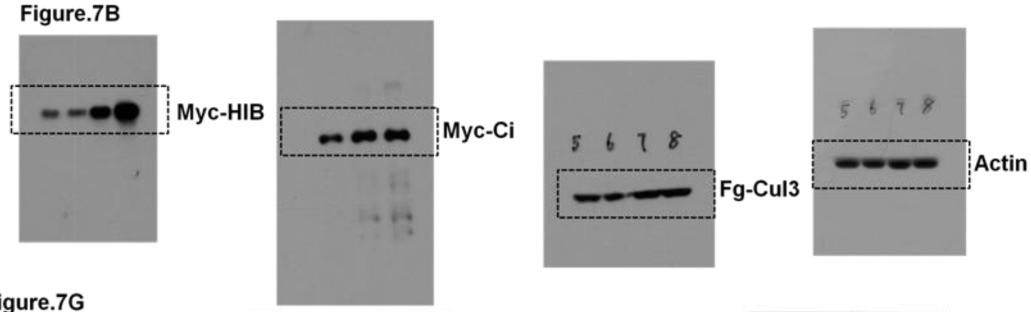

Figure.7G

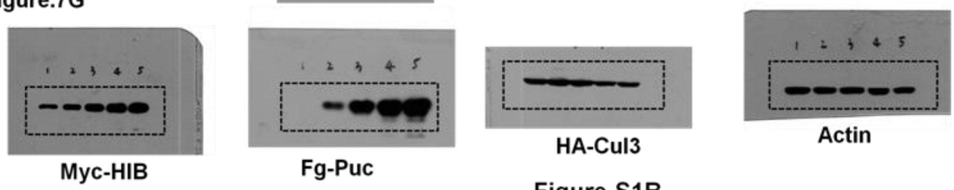

Figure-S1A

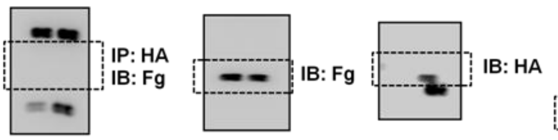

Figure-S1B

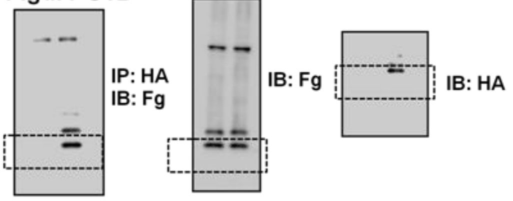

Figure-S1C

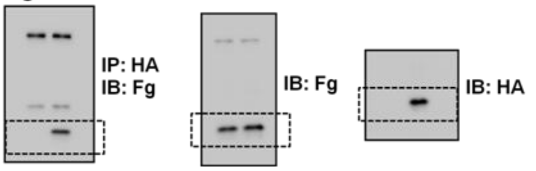

Figure-S1D

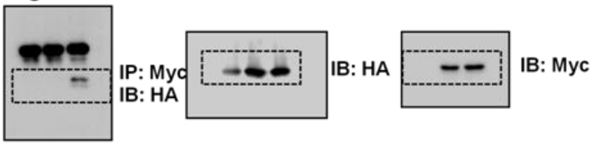

Figure-S2A

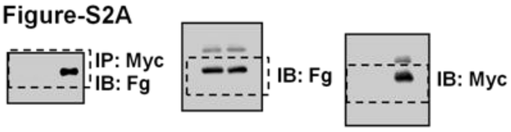

Figure-S2B

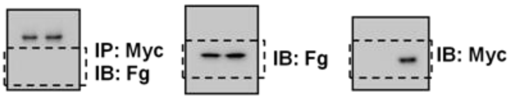

Figure-S2C

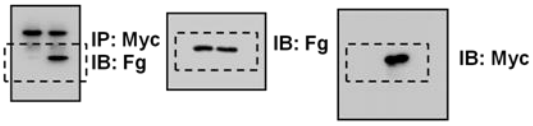

Figure-S2D

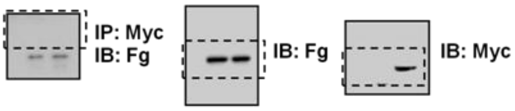

Figure-S2E

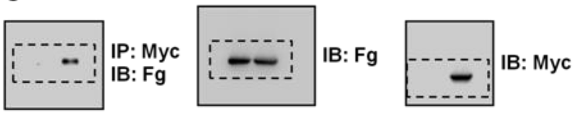

Figure-S2F

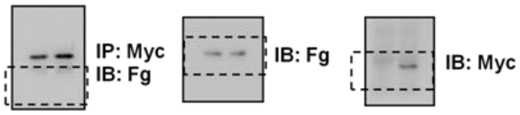

Figure-S2G

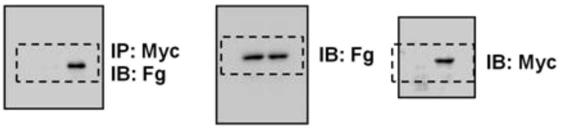

Figure-S2H

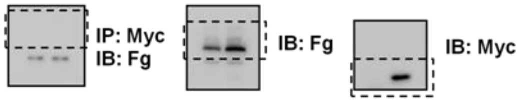

Figure-S2I

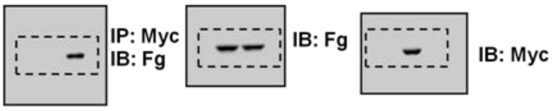

Figure-S2J

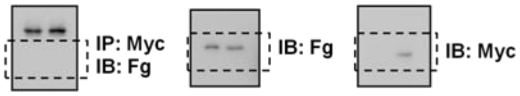

Figure-S2K

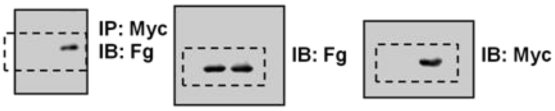

Figure-S2L

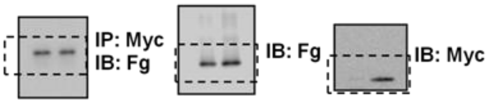

Figure-S2M

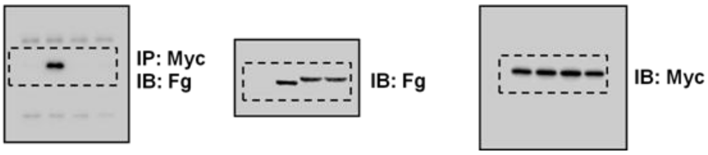

Figure-S3A

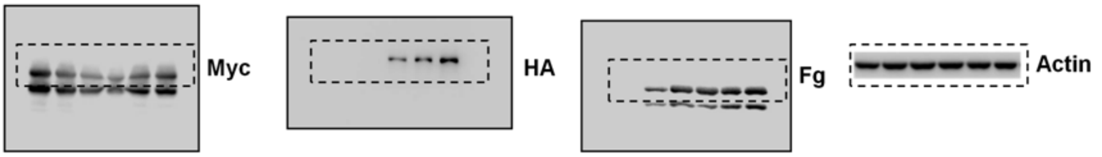

Figure-S3B

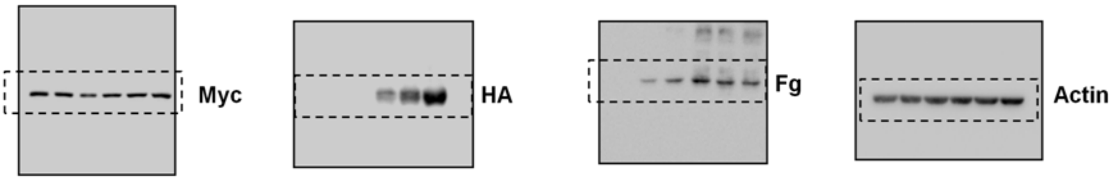

Figure-S3C

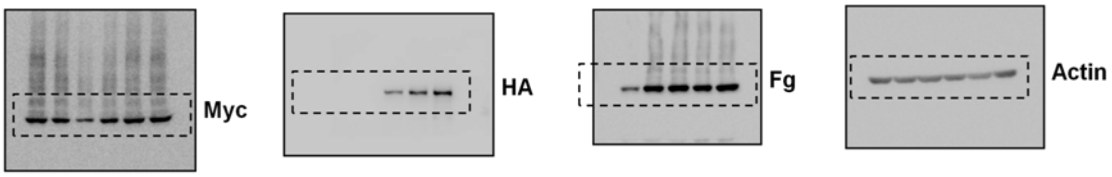

Figure-S3D

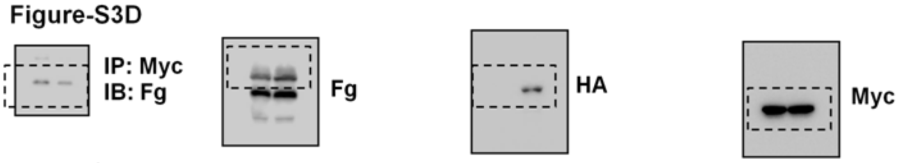

Figure-S3E

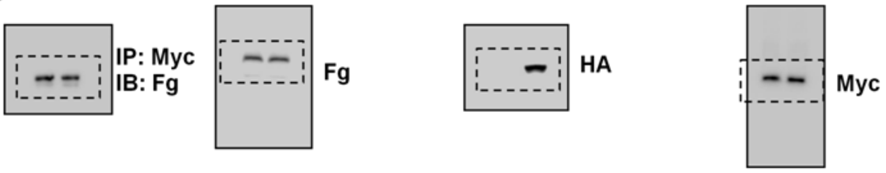

Figure-S3F

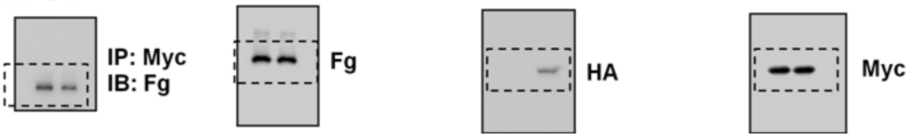

Supplement: Supplementary Information [file srep12709-s1.pdf]
